# Rudolf Virchow1Delivered before the Medical Society of the State of New York at Albany, January 28, 1903.

**Published:** 1903-04

**Authors:** Charles A. L. Reed

**Affiliations:** Cincinnati, O., Former President of the American Medical Association


### SPECIAL ARTICLE.

### Rudolf Virchow.[1]

### An Appreciation.

BY CHARLES A. L. REED, M. A., M. D., Cincinnati, O.,
Former President of the American Medical Association.

THE object of this address, the invitation to deliver which is an honor for which I am profoundly grateful, is to express, in some measure, an appreciation of the life and labor of Rudolf Ludwig Karl Virchow, a deceased honorary Fellow of the Medical Society of the State of New York.

THE ESTIMATE OF GREATNESS.

In approaching this task, we become at once impressed with the fact that the influences which develop greatness are subjects of speculative inquiry, not less interesting and important than the momentous question of what constitutes greatness itself. When, therefore, we for any reason examine into the facts relating to the evolution of a given historic character, we at once think of the conditions and forces concerned in its production—we think of ancestry, of domestic surroundings, of scholastic opportunities, of personal associations, and of the forces that were at the time dominant in the social, political and intellectual atmosphere. We are prone, also, as we turn to greatness itself, to measure it, not alone by the standard of its own time, not alone by the rule of personal achievement, but to estimate it with reference to both its immediate importance and its final influences. Thus, as we glance over the vista of history, and our fancy nestles, naturally enough, about the most imposing

1. Delivered before the Medical Society of the State of New York at Albany, January 28, 1903.

figures of the ages, we discover, for instance, that we would like to be better acquainted with the parents of Julius Cæsar—the man of action; that we would like to know more of John and Mary Shakespeare, who blessed mankind with the Bard of Avon—the man of sympathy; and we yearn for an acquaintance with the farmer of Woolsthorp and his wife who begat the illuminating genius of Newton—the man of mind. We feel, also that, full as are the annals, we would like to know even more of the actual political forces of the Roman Empire, directly concerned in developing the world's greatest soldier and statesman; that, notwithstanding its rich literature, we would like to feel the sentient throbs of the Elizabethian epoch that made possible the world's greatest poet and dramatist; that, voluminous as is the record, we would like to be more familiar with the trend of scientific thought during the century that followed and that produced the world's greatest philosopher. We search for the reason why each of these names has been engrossed upon the scroll of immortality, and as we search we discover that everywhere lies the unknown; that, in atom and planet, in germ and genus alike is law, natural law, inherent and integral, whose essence is not and cannot be of the record; that these men have delved and found and revealed laws,—laws of war and statesmanship, laws of human emotion, laws of the natural universe,—and that man is stronger and better and happier because they lived. We discover, furthermore, as we glance over that immortal scroll, that each name inscribed thereon, has been placed there because its possessor, whether Pythagoras or Euclid, Copernicus or Kant, Galileo or Herschel, Hippocrates or Harvey, whether Dante or Goethe, each has been recorded because each has torn away the veil and revealed somewhat of the law that was hidden; for the law is in all and of all, and he is the greatest among men who reveals the most of law unto man. In this spirit let us approach a discrimination of the great savant whose demise was the melancholy event in the medical, the scientific, and the political world during the last year.

THE YOUTH OF VIRCHOW AND ITS ENVIRONMENT.

The Second Peace of Paris had been signed but a few years when, in 1821, Virchow was born in the little hamlet of Schievelbein in the flatlands of Pomerania. The Baltic breezes that swept inland on that fifteenth day of October were not, however, sufficient entirely to cool the political atmosphere of that northernmost Prussian province, or, for that matter, of the thirty-nine petty states that then comprised the German Confederation. Napoleon's exile had terminated with his death at St. Helena, but six months previously, and Europe,—Germany in particular, —relieved of the depressing shadow of his overlordship, was busying itself with the always serious problems of reconstruction. The intellectual world was not less perturbed than was that of politics. The great universities, then as now, exercised a powerful influence upon the trend of events. It was in them that the battle for constitutional rights, as a remedy against the despotism of the petty princes, was waged with intensest vigor. A constitution had been granted and revoked in Wurtemburg; the Duke of Weimar had granted a constitution to his subjects, the celebration of which event, at Jena, had led to patriotic demonstrations, and to a revival of the influence of Luther's great struggle for liberty of thought. The movement, thus engendered, had become so formidable, as to invoke the oppressive antagonism of the tyranous Metternich, manifested among other things by the promulgation of the infamous Carlsbad decrees, which provided for the rigorous censorship of the universities and newspapers by government commission. Their provisions involved the suppression of any newspaper and the exile of any man who might express opinions inimical to the policy of the government.

The same interference with free thought existed in Austria and Lombardy. The students of the University of Turin had been massacred because they had appeared at the theatre in red caps. France and Spain were in a state of unrest, and in the countries of Europe—the people—from the Mediterranean to the Baltic, the effort was being made to conform human life to those inherent laws of the social fabric that most make for happiness. The effort to reduce these laws, natural, inherent laws, to definite terms; the effort to adjust habits and customs to new ethical rules and to new constitutional provisions, produced a state of mental activity and of moral daring in every part of Continental Europe. This, then, was the social, political and intellectual atmosphere that prevailed in every German home, even in the home of Karl and Johanna Virchow, as they rocked the cradle of him, the formal appreciation of whose long and illustrious life is the object of our solemn reunion at this hour.

The clamor against absolutism was heard in childish murmurs at the public school at Schievelbein, to which young Virchow went at a tender age. There, in the little town in which the Reformation had long been the dominant force; there, in the little school beneath the shadow of the church, the synagogue and the Castle of Malta,—a combination that in its catholicity was almost prophetic,—the youth encountered forces that were potent in fashioning his subsequent illustrious character. It is this fact that we, in free America, where the schoolhouse stands as the temple of rational belief, where it stands as the safeguard of the republic, may take peculiar satisfaction.

### FORMATIVE INFLUENCES OF VIRCHOW'S CHARACTER.

The political agitations of the times, the little rivalries, the little hatreds, the fierce combats of the public schools, were not, however, sufficient to divert the youthful pupil from the successful prosecution of his studies; for, we learn that he went, under age and with a particularly advanced knowledge of Latin, to the gymnasium at Koslin, where he was a source of surprise to the directors. Here at Koslin, again, was the spirit of the Reformation, with its inspiration of truth and liberty, and its yearning for happiness. The fact was recognised even as far north as Pomerania, that, in the Rhenish provinces, previously ruled by French officials, there was a higher idea of human rights than obtained in the other provinces of the confederation, especially under those ruled by the powerful house of Brandenburg. There was, therefore, a clamorous appeal for the recognition of all that was attractive and great in the principles of the French Revolution, and the outcry for a constitution, embodying these principles, came from no province with more emphasis than from Pomerania, and from nowhere in Pomerania with more insistence than from Koslin and from Schievelbein. The revolution of 1830 had brought coveted charters of liberty to Brunswick, Hanover, Saxony and Hesse-Cassel, while to Prussia it had brought only the farcical concession of a system of triennial, provincial diets with merely consultative powers. In spite of these distracting influences, however, influences that are always alluring to the enthusiasm of youth, young Virchow passed from the gymnasium in 1839, first on the list of the *Abiturienten*. The independence by which industrious and ambitious youth refuses to be restrained within the confines of an arbitrary curriculum is always the prophecy of a broad manhood. The child, in this instance and by this rule, was, indeed, father to the man, for we find that he presented himself for his finals, not only in the required branches, which were difficult enough, but in Hebrew, which he had mastered from pure love of philologic research. It was this same impulse that prompted him, during the succeeding few months, to master Italian without a teacher, just as, years later, we find him resting himself from his scientific labors by delving into the charms of modern Arabic poetry.

### VIRCHOW AND JOHANNES MÜLLER.

A few months after leaving the gymnasium, he set out for Berlin, a journey, which, in those days, before the introduction of railroads, had about it more of adventure than is involved in the two hours' run of today. Of his career as a student in the Friederich Wilhelm Institute, it is sufficient to say that he was an arduous student. In the faculty then were Dieffenbach, the foremost surgeon of the day; Schönlein, who had come from Zurich the same year to join, not only the teaching body, but to act as reporting council for the ministry and to serve as physician-in-ordinary to the King; Froriep, who was in charge of the Pathological Museum at the Charité, and who, in addition, served the government as medical counsellor; Casper, who was also a medical counsellor, with a seat in the medical deputation for medical affairs in the ministry; but, towering above all was the intellectual figure of Johannus Müller, the professor of physiology. He was an original genius, with daring, actually engaged in winnowing the wheat of demonstrable truth from the then prevailing chaff of egoistic opinion,—to divorce a physical science from speculative philosophy. Prompted by the inspiration which he in turn had derived from Bichat and the French School, this professor of physiology was busily engaged in retesting in the laboratory truths previously elaborated by Haller, Whytt, Spalinzani and Bichat himself.

My fancy likes to dwell upon the almost dramatic moment when the shopkeeper's son from Schievelbein, the little keen-eyed, yellow-haired stripling of 19, was ushered into the presence of this, the great founder of the modern school of physiology. There was in that meeting an intellectual impact that resulted in the transference and the perpetuation of great thoughts, great methods, which, perfected by the pupil, led to still greater results. It was from this great professor that Virchow, during the next four years was to derive those habits of investigation which, coupled with the spirit of daring, was to make him in turn the leading investigator in the realm of biological research.

It must be remembered, however, that with all its social and political disturbances, Germany was at that time thoroughly impregnated with a wholesome ferment. It consisted of the spirit of rational investigation, and was infused by Liebig in chemistry; by Humboldt, who was promulgating his discoveries leading to the publication, five years later, of his Cosmos; and by Froebel, who was establishing his marvelous principles of education derived from Pestalozzi, and which have since borne rich fruit the world over in every department of human instruction.

VIRCHOW'S EARLY RECOGNITION.

It is not surprising, therefore, that, with these antecedent influences, with these present surroundings, with these dominating forces, and with his marvelous insight and industry, Virchow should make such a record as a student, that upon his graduation he was given the assistantship to Froriep, the Prosector of the Charité Hospital. It was his first recognition, and it came with deserved promptitude.

He was actuated at this time, as in his entire subsequent career, by the broadest principles of catholicity. During his student career, in addition to the prescribed lectures, he had gone into logic and psychology; in his busy energetic way, he had mingled with the political organizations among the students, and there were already manifest tendencies which, a few years later, brought him before the German public as a scientist, a philologist, a social reformer, and a democrat. Promotion came without undue delay. Froriep resigned as Prosector in 1846, and Virchow was elected to the succession. His work with Müller, however, had brought him in contact, not alone with that great man's scientific method, but with his habits of publicity, as a scientific writer. The *Archiv. f. Anatomie, Physiologie u. wissenschaft Medicin*, long issued by Müller, soon found an imitator in the department of pathology in the periodical issued jointly by Virchow and Reinhardt, and which, on the demise of the latter, Virchow continued to edit until his death. The political and economic conditions were fashioning themselves into the Revolution of 1848, when Virchow, already in influential touch with the Prussian Government, was delegated to investigate an epidemic of typhus fever which was then raging in upper Silesia. The work was done with his characteristic thoroughness, transcending the prescribed limit of his instructions. He not only investigated the pathological and clinical phases of the disease, but he entered freely into a discussion of the hygienic, economic and social conditions underlying the epidemic. He even went so far as to indicate a number of social reforms, essential to the prevention of such epidemics, and tinctured his science with considerable democracy, his outspoken utterances in these particulars causing a distinct sensation in the ministry.

### VIRCHOW'S POLITICAL ACTIVITIES AND HIS BANISHMENT FROM BERLIN.

The trip to Silesia seems to have been a very important experience in Virchow's career. His previous tendencies as a reformer, in the direction of popular liberty, were now fully confirmed, and he became an active participant in the great revolutionary movement of that year. He unhesitatingly promulgated his platform as that of full and unrestricted democracy, on which theme he made violent speeches to the Berlin populace by whom he was elected a member of the national assembly. His political ambitions, however, were destined to be temporarily curbed by the fact that he was under the parliamentary age, and was, consequently, not permitted to take his seat. His energy, however, found a compensatory outlet, for, with Leubuscher, he founded a journal which they called *Die medicinische Reform*, through which he advocated the establishment of a ministry of health, and insisted, among other measures, that medical education should be made free. These suggestions, not originating with the government, were scarcely less distasteful to the ministry, than was his report from Silesia, or than were the political harangues which he continued to pronounce to the plaudits of his fellow burghers. He seemed at this time to be largely dominated by the spirit of iconoclasm, not, however, that form of iconoclasm which is merely an expression of the spirit of destruction, but that better iconoclasm by which old gods are destroyed that newer and better ones may be erected. He invaded the realm of theology and proclaimed, not a mere agnosticism, but a positive disavowal of the existence of a Hell, insisting that "only a benighted Mecklenburg pastor could be so foolish as to believe in a Devil." The government, committed not only to the task of maintaining the National order, the National laws, but the National religion, looked upon the young orator as a dangerous polemic. He was, accordingly, compelled to resign his appointment at the Charité, where, in spite of all his political agitations, he had conducted epoch-making researches on leukemia, embolism, thrombosis, phlebitis, and other phases of morbid anatomy. He had already become a teacher of ability, and his researches had attracted widespread attention. These facts, quite as much as the influence of his colleagues at the Charité, probably saved him from the decree of exile, issued at that time against many participants in the Revolutionary movement. He was, however, banished from Berlin to Würzburg, where, in May, 1849, he accepted a chair in the faculty of the university. There was here less opportunity for effective participation in the political movements, and his energy found fuller exercise in the prosecution of his original researches, and in the exercise of his philological tastes.

### ACTIVITIES AT WÜRZBURG.

During the seven years that he remained here he kept up his study of Italian, Arabic, and acquired a knowledge of English. His scientific researches at Würzburg embraced the subjects of phthisis, tuberculosis, typhoid fever, cretinism, hydronephrosis, adipocere, echinococcus of the liver, amyloid degeneration of lymphatic glands, the corpuscles of bone, cartilage and connective tissue, and he thoroughly investigated the anatomy of the nails and the epidermis. *Die medicinische Reform* was discontinued shortly after he went to Würzburg, but the young professor, instead edited a *Handbuch der speciellen Pathologie*, and, in connection with J. Vogel, issued a manual of general pathology.

### RECALL TO BERLIN.

It seems from careful study of Virchow's career that it was at about this time that his observation of concrete facts had become sufficiently extensive to justify him in venturing upon important generalisations; for the little manual issued in connection with Vogel contained many of the fundamental principles which, a few years later, were elaborated into his *Cellularpathologie*, published in 1859. He had been recalled to Berlin in 1856, under circumstances that invested the incident with the characteristics of a triumph. The chair of general pathology had become vacant through the resignation of his former teacher, Froriep. In all Germany there was none so able to fill it as the young democratic professor. He was sent for—but paused to consider. When his reply came it brought his acceptance, based, however, upon the condition that an institute for practical work should be founded. His terms were accepted, not only in this, but in other particulars, and he at once entered seriously upon what must be recognised his more distinctive life work. The Museum at that time contained 1,500 preparations; at his eightieth birthday, as the result of his own individual labors, the number had increased to 23,000. In his work he was actuated by the view, expressed in his own words that, "the rôle of pathological anatomy as a dogmatic science is at an end; for each individual law we must have the proof, clearly recognised and carrying personal conviction." He insisted that the whole of the then existing system must be abolished, and that a new philosophy, based upon observation and experiment, must take its place. This new pathology, he insisted, must come about gradually, and not as the mental product of individual enthusiasm. It must be achieved as the outcome of laborious research by many competent investigators, and, when thus evolved, and thus only, could it be accepted as the basis of scientific medicine.

### VIRCHOW AS A LEGISLATOR.

The engrossing character of Virchow's labors at the Institute at this time, the absorbing enthusiasm involved in the promulgation of a new and revolutionising philosophy, the exactions of editorial duty, all combined with the responsibilities of professorial work, were not sufficient, however, completely to divert his attention from collateral and often apparently irrelevant studies, and from participation in the fierce political controversies that were then agitating the German people. William I. had ascended the Prussian throne in 1858. There was some hope of relief from the oppressive measures of his predecessors, and this very hope stimulated the activities of the Democrats, or of the "Demogogen," as the party was opprobriously designated by the Conservatives. Virchow, notwithstanding his unpleasant experiences that had resulted from his banishment to Würzburg, immediately identified himself with the cause of popular liberty. In this he was actuated by a profound contempt for the reigning house, a contempt which, on occasion, found expression in his famous observation on heredity. "I know a family, a very exalted one," he was wont to say, "in which the grandfather had softening of the brain, the son hardening of the brain, and the grandson no brains at all," the reference being to the three Frederics, the immediate predecessors of the then reigning monarch. The work of the mere agitator, however, was not sufficient for one of Virchow's temperament, particularly to one who, after a previous election to the National legislature, had been denied his seat on account of his youth. He was, in 1862, older by fifteen years, and, accordingly, offered himself as a candidate for the Prussian chamber, to which he was duly elected. It was in the same year that Bismarck became Prime Minister, a coincidence which marked the beginning of an antagonism that continued throughout the political careers of the two men.

Virchow speedily became the leader of the Radical party, and by his advanced views and cogent reasoning, and by his courageous insistence upon them, speedily earned for his policy the opprobrious designation of "Professorismus," applied by the Iron Chancellor. While these debates were going on, the duties at the Institute, at the Charité, and at the editorial office were not neglected, although there is ample testimony that Virchow was often tardy in keeping his appointments. The famous Schleswig-Holstein episode diverted for a time the attention of the legislature from internal to external affairs, and culminated in the war with Denmark in 1863. In this war, Bismarck, against Virchow's opposition, used Austria as the cat with which to pull the chestnuts from the fire, and then, three years later, again over Virchow's opposition, he proceeded to kill the cat. As a result of this war with Austria in 1866, the Germanic Confederation of 1815 was terminated, and the North German Confederation took its place. It may be premised that Virchow, who, with all his democracy, was always a Unionist and a Nationalist, deprecated this segregation of the Germanic people.

### VIRCHOW AND BISMARCK.

It was in the course of this long sustained opposition to the policy of the Government that he, as chairman of the finance committee, a position which he held for many years, succeeded in defeating an appropriation for naval purposes that had been demanded by Bismarck, who, thereupon, challenged his successful antagonist to mortal combat. Virchow, with no disposition, whatever, to give the Herculean warrior an opportunity to exercise his professional skill, and with moral courage to stem the tide of sentiment in favor of duelling that still disgraces Germany,—a courage vastly excelling mere physical bravery,—

declined the cartel, but continued his opposition. This opposition was carried along through the days of the Franco-Prussian War, but when the first shot had been fired, Virchow, always a patriot, and always the physician, took his son and joined the army, the two serving in the capacity of surgeons in the field. These men, father and son, did their full measure of duty, conspicuously upon the field of Metz, in alleviating the sufferings of their wounded compatriots. No sooner, however, had peace been concluded with the proclamation of William I. as Emperor, at Versailles, than Virchow resumed his wonted activities in science, in literature, in politics at Berlin. It was then that, probably for the first time in his political career, he found himself *en rapport* with the leading features of Bismarck's policy—namely, the policy that involved the construction of the German Empire. It may have been this particular fact, quite as much as a general appreciation of Virchow's work, that prompted Bismarck, before his own retirement under the present Emperor, to apologize publicly for many asperities which had characterised his previous attitude toward the great savant.

About this time the widened scientific view of Virchow, a view which had come to embrace the whole science of man, as comprehended in the then slumbering science of anthropology, began to be manifested in his contributions to literature. He was already accumulating facts which were to serve as the ground work of ethnology, yet, in spite of all this, acting in his capacity as a member of the Town Council, a position which he held for more than forty years, he was not oblivious to the fact that the sanitary condition of Berlin was deplorable. He, accordingly, became responsible for the establishmnt of those enormous hygienic reforms that have banished typhoid fever and other zymotic diseases from Berlin, and that have rendered that city one of the most salubrious in the world.

### LATER WORK IN ARCHEOLOGY AND ETHNOLOGY.

Archeology, also, was at this time engaging his attention, and in the midst of the preparation of his valuable work on *The Topography of Troy*, he, in 1878, retired from active political life, only, however, to be elected two years later to the German Reichstag. In this body, however, he was always an interested spectator, rather than an active participant, and never aspired to the office of party leadership. It is not to be assumed, however, from this that Virchow's intellectual activity was by any means at an end, or even upon the wane. The twenty years following his election to the Reichstag were among the most fruitful, intellectually, of his entire life. He amplified in many particulars his teachings of cellular pathology. He delved more deeply than ever into the hidden mysteries of ethnology, producing, in 1882, his valuable work on *Old Trojan Graves and Skulls*. At the very pinnacle of scientific fame he kept himself *au courant* with the whole trend of scientific thought, delivering an address before an International Congress at Berlin, at Paris, at Moscow or at Rome, laboring in an assemblage of scientists here, or in a hygienic congress there, or delivering a Croonian or a Huxley lecture in London. With his editorial labors always in hand, he still clung industriously to his old haunts in the Pathological Institute, in the Anthropological Museum, or in his ward at the Charité; for, be it remembered, Virchow was always a practical physician. It has been said of him that during these years he knew no such thing as a vacation, in the ordinary sense of the word, for it was his habit rather to find recreation in a change of occupation, such, for instance, as visiting Asia Minor, and, pick in hand, to assist his friend Schliemann in his wonderful archeologic researches.

### THE HUMAN SIDE.

In the midst of it all he was very much of a man on the human side, a little wiry man, but a little over five feet in stature, sprightly, congenial, loving and lovable. His domestic life has been described as ideal. The many Americans who were present at the Berlin meeting of the International Medical Congress, will recall his active and whole-souled participation in the festivities of that occasion. He was given a Festschrift on his seventieth birthday, and again on his eightieth, on which later occasion, in particular, delegates were present from practically every country, and festivities were held simultaneously in practically every leading city of the world.

On January 3, 1902, he sustained a fracture of the neck of the femur by falling from a tram car. He died September 5, 1902, mourned by the civilised world. The municipality of Berlin, which he had faithfully and efficiently served as a councillor for so many years, accorded him the distinction of a public funeral, which, in the midst of universal mourning, was participated in by many officials from the political and scientific world.

### GERMAN MEDICINE AT THE BEGINNING OF VIRCHOW'S CAREER.

This, then, was the man upon whose work we are called, at this hour, to pronounce a formal appreciation. It is rare, indeed, that the occasion arises to attempt, in even a desultory way, the estimation of a career that has resulted in the establishment of two distinct, although correlated sciences, and in the substantial advancement of human liberty. It would be quite out of the question in an address such as this, to attempt a résumé of his doctrines in pathology, a mere enumeration of his contributions to which would involve the employment of more than twice as many words as I shall employ in your hearing. We may, however, arrive at some estimate of his work in this great department by pausing, for a moment, to consider the state of medical science, or more particularly, the conceptions of disease that obtained in Germany when Virchow was made the successor of Froriep at Berlin. It is true that Rokitansky had introduced many of the revolutionising doctrines of Bichat at Vienna, but even Rokitansky was busying himself to an important extent in promulgating the purely dogmatic doctrine of crases.

Oken, at Munich, was indulging in the glittering generality that life is the self-generation of individualised elements, that the principle of life is galvanism, and that vital force is galvanic polarity. Of him Agassiz declared that he constructed the entire universe out of his brain. Dollinger, of Würzburg, the father of the great theologian, belonged to the same speculative school which a historian has designated as the "Romanticist or Teutomaniacs." At Berlin, Schönlein, who had been one of Virchow's teachers, and was yet his colleague, and who represented what was designated as the Natural History School, taught that disease was an entity, a sort of parasite, sojourning temporarily in the body, just as Paracelsus had once spoken of a "microcosm within a microcosm." Schönlein, more specifically, looked upon disease as a sort of equivocal infusoria, the existence of which he logically predicated, but never, of course, physically demonstrated. These infusoria, the existence of which was thus gratuitously assumed, were easily enough imagined to consist of genera and species, each producing different clinical phenomena—a sort of empirical prophecy of the germ theory which has since played so important a rôle in medical philosophy. It was against such theoretic doctrines, then dominant, that Virchow brought the evidence of the microscope and the revelations of mortuary. He began in the truly scientific manner, which consists always, first in the observation of concrete facts, next in their classification, and third in their ultimate generalisation.

### THE DOCTRINE OF VIRCHOW.

His labors at Würzburg, supplemented by those conducted under more favorable auspices, after his return to Berlin, enabled him to announce as the seminal doctrine of his philosophy,—and I employ the words he subsequently used at the fiftieth Congress of Naturalists and Physicians,—namely, that the new science was based "chiefly on the recognition of the fact that the cell is actually the ultimate, proper morphological element of every vital manifestation—*omnis cellula e cellula*—and that we must not remove the proper action beyond the cell." In the early elaboration of this doctrine, taking up the work where Schwann and Schleiden had left it, he early proclaimed the importance of the nucleus to the maintenance and multiplication of the cell, and emphasised the fact that tissue growth implies cell multiplication, while the contents of the cell, and even of the material deposited outside of it are of controlling importance to function. He taught, furthermore, and as a necessary corollary of the preceding postulates, that tissues vary in function according as they vary in cellular construction. He insisted upon the existence of an intercellular tubular system that supplemented the recognised circulatory systems in the work of ultimate nutrition.

As a result of his investigations of the circulatory apparatus and of the blood, he taught that the walls of the bloodvessels were impervious and argued that blood, or even the nutrient elements of the blood, could not escape from them without rupture of the wall, which rupture was, however, rarely, if ever, demonstrable. It would seem that in this doctrine, which I believe is as near an approach to empirical dogmaticism as could be found in all his teachings, Virchow laid a logical foundation for the new doctrine of osmosis, that today promises to take both physiology and pathology largely into the realm of physics. He directed his arguments specifically against the then prevailing humoral pathology by insisting that the blood itself is not the proper and original cause of dyscrasiæ, as taught even by Rokitansky, but that instead these dyscrasiæ have their origin rather in a disturbed metabolism, the toxic products of which are merely carried in the blood. These laborious studies of many phases of blood changes, comprise the basis of our present accurate conception of the pathology of the circulatory medium. His study of inflammation, in the description of which he insisted that disturbed function should be added to heat, pain, redness and swelling, as one of the cardinal indiciæ of the phenomena, gave an accurate conception of the actual changes. His elaborate investigations of the nervous system resulted in the promulgation of doctrines whose parentage in the works of Brown and Haller is recognisable. His work on tumors, a distinct application of the cellular doctrine, stands today as the fundamental classic of the subject. His investigation of tuberculosis resulted first in his classification of the disease into neoplastic and inflammatory forms, but laterally he recognised the bacillary forms.

### THE POSITION OF CELLULAR PATHOLOGY.

It would be impossible, however, as I have before stated, to give even an accurate résumé of this extensive philosophy, the application of which to the entire phenomena of disease must stand as his crowning achievement. It is interesting to hear him recount, as he did in a lecture delivered in London, during the last years of his life, the general summarisation of his work in the statement that "the law of continuity of animal development is, therefore, identical with the law of heredity, and this I was now able to apply to the whole field of pathological new formation." And it was especially interesting, in view of the ideas against which he had to contend, to hear him add with pardonable exultation: "I blocked forever the last loop-hole of the opponents, the doctrine of specific pathological cells from which types and ancestors were not forthcoming in normal life."

The doctrines which he had thus established, and to which he thus alluded, became, early in their history, the actuating principles of the "Berlin School," which, sooner or later, embraced the names of Leyden, von Rechlinghausen, Cohnheim, Waldeyer, Hoppe-Seyler, Kuhne, Rindfleisch, Klebbs, Liebrich, Frederic, in Germany; Felix Simon, in England; and, conspicuously W. H. Welch, in the United States. The principles taught by this school are, by common consent, those upon which modern surgery and rational therapy alike are based.

The position that must be accorded this doctrine in the light of further revelation of fundamental law, cannot be foretold.

Nothing could be further from the purpose of Virchow himself, than the assumption that his doctrine represented the finality of truth or that it made impossible further discoveries either based upon or antagonistic to it. Much has, indeed, been made possible since the date of his earlier revelations. This is notably true of the doctrine of the infectiousness of disease, which is based upon observations that were made possible only by a later perfection in optics and by a later advancement in the technique of biologic research. The most that can be said of the relation of the germ theory of disease to that of cellular pathology is, that without invalidating the important conclusions embraced in the latter, it left Virchow's recorded observations unimpaired and undisputed. The new doctrine of the ions, involving the principle of osmosis, may bring other and important supplementary facts which shall serve to show that the discoveries of Virchow comprised in the aggregate a single, but important link, in the evolving chain of science.

### VIRCHOW AND THE FOUNDATION OF ETHNOLOGY.

The next phase of Virchow's character as a scientist relates to his work in the department of anthropology. This, the science of man in its broadest conception, can scarcely be said to have had more than a mere commencement before Virchow, beginning with his work in biology, was led into it by the widening circle of associated ideas. It may be said, indeed, no valuable contributions were made to the subject during the first half of the 19th century. Blumenbach, of Göttingen, had made his famous collection of skulls,—his "Golgotha" as he called it,—which was the basis of his own investigations, and which may be said to have been the starting point of systematic anthropological study. About the same time, that is the last years of the eighteenth and the first years of the nineteenth century, von Sömmering, of Frankfurt, studied the eyes, not only with reference to their anatomical detail, but with reference to their ethnic significance, while Camper, of Holland, made a careful study of the facial angles. This was practically all that was done with the subject, until Darwin issued his *Origin of Species*, in 1859; his *Descent of Man*, his first contribution to the subject of ethnology, did not appear until 1872. Long before the latter date, however, Virchow had taken up the subject at two points of contact. The first point of contact was developed out of his philosophy of cell genesis, the doctrine that all cells are derived from preëxisting cells, which he promulgated in 1859, and which brought up, as a natural corollary, the question of variation of type. His antagonists,—the believers in special creations,—seized eagerly upon this declaration as a refutation of the then rapidly growing materialistic philosophy, and as a vindication of their own ontologic dogmas.

If the cell is the vital unit, as Virchow declares, and if the individual is but the sum of cells, they urged, then variation in the individual can occur only as the result and commensurately with the variation in the constituent cells; if, they added, like cells always beget like cells, as Virchow declares, then the individual, the sum of cells, cannot vary from his cellular type; and, finally, they insisted, if all the cells in the individual have been derived through the generations from cells of the same type, then the original cells, at the beginning of things, must have been the products of a special and miraculous creative act.

Unfortunately, however, for this specious logic, Virchow taught, in effect, that like cells begat like cells only, however, under like circumstances, and that, as the circumstances vary, so does the cell type vary. This is, indeed, the point of departure from the standard of health, the very beginning of pathological phenomena.

### VIRCHOW AND DARWIN.

As a matter of fact, Virchow simply declined to discuss the origin of species until sufficient evidence to justify him in doing so could be derived from a careful search of the tissues. He recognised the mutability of the cells, and realising, logically, that variations in type must begin in these vital units, he, without denying the truthfulness, or affirming the falsity of Darwin's hypothesis, simply awaited the demonstration of the actual changes within the cell. It is an interesting fact, and one bearing testimony to Virchow's scientific acumen, that this very variation was reduced to a physical demonstration in 1900 by Professor Guyer, of the University of Cincinnati, whose investigations are recorded in his valuable contribution on "Hybridism and the germ-cell." It is also of striking interest, at this time, and one bearing testimony to the reliability of Virchow's deduction, not only that these observations of Guyer's, but that Mendel's law, promulgated through an obscure periodical at Brunn, Austria, in 1865, seemed to cover the entire point. This law of Mendel, or, as I believe we should now call it, the Mendel-Guyer law, is in effect that, as a result of definite and demonstrable changes in the germ cell, the second and later generations of a hybrid possess every possible combination of apparent characters, and that each combination appears in a definite proportion of the individuals, the whole reduced to the terms of a definite equation. This law, revealed by observations in both the animal and vegetable world, seems to be one of general applicability, and one that is calculated to invest the conclusions of Virchow with an increased value.

The next point at which Virchow was brought in contact with the general problem of anthropology, or more particularly, that of ethnology, grew out of his studies of cretinism, and of the causes of variations in the growth of the skull. It was precisely this study of the pathologic phases of craniology that enabled him to detect morbid changes in the celebrated Neanderthal skull, which, with its protruding supraorbital ridges, its low forehead, and its small cranial capacity, even the scientific world was too disposed to accept as the normal index of a racial type that had long since passed away. Virchow further insisted that, even if it were normal, the existence of a single skull was not sufficient evidence upon which to predicate the existence of an entire race, and that conclusions should be withheld until further evidence was secured. It was this cautious utterance, thoroughly characteristic of Virchow, that gave the theologic polemics another opportunity falsely to proclaim that he was an antagonist of the doctrine of descent, as promulgated by Darwin. It seems that the utterance, seized upon for this particular misrepresentation, occurred in an address delivered in 1877, before the German Naturalists and Physicians, and was to the effect that the hypothesis of Darwin ought not hastily to be given the force of law,—that it ought not to be placed in the category of law,—without first waiting to gather and accumulate all relevant facts. It was just this scientific discrimination between hypothesis and law—a discrimination repeatedly expressed and emphasised by Darwin himself—and just this conservative tendency in the consideration of demonstrated facts, and in the formulation of conclusions based upon them, that gave to the judgment of Virchow the greatest possible weight in the scientific world. And it was this very weight which he, himself, as late as 1900, with true scientific spirit, was disposed to deprecate; for he had spent his life in dethroning the power of personal influence, and in establishing the regnancy of demonstrated truth.

TENTATIVE CONCLUSION IN ETHNOLOGY.

His work in anthropology, however, considered from its positive side, was very great. He was always an organiser,—a valuable weakness in a man of brains,—and it was by this means that much of his work was brought to its full fruition. He organised, or at least assisted in the organisation of the German Anthropological Society and the *Berliner Gesellschaft für Anthropologie, Ethnologie und Urgeschichte;* he helped to found the *Museum für Volkstrachten;* and the almost invaluable *Archiv für Anthropologie.* He, with his colleagues, gave serious study to the physical characteristics of the early Germans. This was supplemented by statistical investigation of the present distribution of the color of skin, eyes and hair in Germany, the whole being reduced to cartographic representation. His descriptions of American crania, based upon Morton's great work, opened that mine of information to German thought. He was the friend and promoter of Schliemann, in whose archeological explanations he was at times a personal participant; he recorded the results of these labors in two books, *Contributions to the Topography of Troy*, and *Old Trojan Graves and Skulls*, each of which is recognised as a valuable contribution to the subject. Extensive, however, as were the researches, and important as were his recorded observations, it does not appear that he considered either of them sufficiently extensive to warrant him in arriving at important general conclusions. He felt justified, however, in saying, as he did say, that physical types do not vary with variations in language and culture, and that different types may blend in the formation of a homogeneous people. This lesson was taught him by a study of the racial types in Germany, and is of extreme interest to us in the United States, where, at this present moment, we are in the midst of the greatest ethnic experiment in the history of the human race. In viewing the entire scope of Virchow's labors in anthropology, it must be concluded that he did not carry them to the point of even relative finality, that he did his labors in pathology; his researches, his discoveries in ethnology must be recognised as fundamental, their true significance remaining to be interpreted in the light of rapidly accumulating evidence. It is sufficient, however, for the perpetuity of his fame, that, by common consent, he is recognised as the veritable founder of this new science, which promises so much for the interpretation of the racial types of men.

### THE FORCE OF THE MAN.

The third side of this great character was the human side, manifesting itself, not alone as a husband and father, but conspicuously as a citizen. He early showed that the prevalent opinion, that to be highly intelligent on one subject it is necessary to be correspondingly stupid on all other subjects, is but a vulgar notion, and he speedily demonstrated that the view-point of the physician is eminently calculated to afford an intelligent insight into social, economic and political conditions. I must however leave it to the political historian and to the public economist to tell what good has been accomplished in Germany in the last fifty years by the liberal movement, a movement that for many decades enjoyed the distinction of Virchow's leadership. A few things are certain: the hated Carlsbad Decrees could not be enforced in Germany today; there is a greater freedom of thought and, what is more important, of expression in German universities than ever before; the offense of *lèse majesté*, strange sounding to republican ears, has a less severe meaning in Germany than it had fifty years ago, and it is equally certain that, for the first time in history, the entire *Vaterland* has a reasonably liberal constitution, wrested from the tyranny of absolutism,—a condition that leads to the hope that the German people may some time enjoy the same beneficent government that today blesses the Great Republic. In the achievement of these results it cannot be denied that Virchow played a leading and an honored part.

### THE FINAL VIEW.

What, then, are we to say in final review of this great man? His figure is that of a colossus, and it will require the perspective afforded by receding years to measure its relative height. Some things, however, we can now tell. He inherited honest blood; he responded in the fullest and in the best sense to the formative influences with which his early life was surrounded; he had the independence to defy personal dictum, and to give allegiance only to demonstrated truth. He had the intelligence to discern and the human sympathy to appreciate that human happiness depended first and chiefly upon a knowledge of the laws underlying and governing human existence; he worked on independent lines and revealed laws of disease previously hidden; he, by his observations and deductions, and by the elaboration of rational methods, laid the foundation of modern medicine; he established the study of racial man as a science; he fought the battle for human liberty, and won for others the boon that he had always arrogated to himself. He added years to the generation of man, and brought happiness to his kind. Finally, let it be recorded, that, above all, he lived faithful to his ideals, —and the greatest of these was Truth.

